# Dosimetric characterization and use of GAFCHROMIC EBT3 film for IMRT dose verification

**DOI:** 10.1120/jacmp.v14i2.4111

**Published:** 2013-03-04

**Authors:** Valeria Casanova Borca, Massimo Pasquino, Giuliana Russo, Pierangelo Grosso, Domenico Cante, Piera Sciacero, Giuseppe Girelli, Maria Rosa La Porta, Santi Tofani

**Affiliations:** ^1^ Azienda Sanitaria Locale TO 4 S.C. Fisica Sanitaria Ivrea (TO) Italy

**Keywords:** GAFCHROMIC EBT3, radiochromic film dosimetry, IMRT verification

## Abstract

Radiochromic film has become an important tool to verify dose distributions in highly conformal radiation therapy such as IMRT. Recently, a new generation of these films, EBT3, has become available. EBT3 has the same composition and thickness of the sensitive layer of the previous EBT2 films, but its symmetric layer configuration allows the user to eliminate side orientation dependence, which is reported for EBT2 films. The most important EBT3 characteristics have been investigated, such as response at high‐dose levels, sensitivity to scanner orientation and postirradiation coloration, energy and dose rate dependence, and orientation dependence with respect to film side. Additionally, different IMRT fields were measured with both EBT3 and EBT2 films and evaluated using gamma index analysis. The results obtained show that most of the characteristics of EBT3 film are similar to the EBT2 film, but the orientation dependence with respect to film side is completely eliminated in EBT3 films. The study confirms that EBT3 film can be used for clinical practice in the same way as the previous EBT2 film.

PACS number: 87.56.Fc

## I. INTRODUCTION

The most currently used approach to IMRT delivery quality assurance consists in delivering the IMRT plan to a phantom and then comparing the 2D dose distribution calculated by the treatment planning system (TPS) with the value measured by radiochromic films that have high spatial resolution and offer the benefit of being self‐developing.

GAFCHROMIC EBT film, released in 2004 by International Specialty Products (ISP, Wayne, NJ), was the first type of radiochromic film suitable for the use with doses as low as the typical doses occurring in radiation therapy. In 2009, the GAFCHROMIC EBT film was replaced by the GAFCHROMIC EBT2 film that incorporates a yellow marker dye in the active layer and a synthetic polymer as the binder component. Several works have been published studying some EBT and EBT2 properties, such as film homogeneity,^(^ ^1^ ^,^ ^2^ ^)^ scanning orientation dependence,^(^ ^3^ ^–^ ^7^ ^)^ energy dependence,^(^ ^4^ ^,^ ^8^ ^–^ ^10^ ^)^ absorption spectra,^(^ ^11^ ^)^ postcoloration behavior,^(^ ^2^ ^,^ ^4^ ^–^ ^5^ ^,^ ^7^ ^–^ ^8^ ^,^ ^12^ ^–^ ^13^ ^)^ high‐dose dependence,^(^ ^7^ ^)^ temperature dependence,^(^ ^7^ ^,^ ^14^ ^–^ ^15^ ^)^ and ambient light sensitivity.^(^ ^7^ ^)^

In 2011, ISP released a new film generation, the GAFCHROMIC EBT3 film. According to the producer's note,^(^ ^16^ ^)^ EBT3 film is made by laminating an active layer between two identical polyester layers, which makes the product more robust and allows water immersion. While the active layer composition and response is unchanged, the real EBT3 improvement are: the symmetric structure that will avoid the potential errors in optical density measurements due to scanning side in EBT2,^(^ ^6^ ^)^ the matte polyester substrate that prevents Newton's Rings formation, and the presence of fiducial marks that allows for the film automatic alignment.

IMRT QA is one of the main target applications of EBT3. However, implementation of EBT3 film for IMRT dose verification has not been addressed in the literature and few data can be found about the new dosimetric properties beyond the manufacturer's product specifications. In particular, Reinhardt et al.^(^ ^17^ ^)^ measured EBT3 response to photon and proton exposure, analyzing film uniformity, dependence on film orientation, and postexposure coloration. This study aims to investigate the most relevant features of this film, focusing attention on its application to IMRT QA, in combination with a flatbed document scanner, and comparing the results with EBT2 film as a reference.

## II. MATERIALS AND METHODS

### A. GAFCHROMIC EBT3 film

The film used in this study was GAFCHROMIC EBT3 (batch number A10171102), with sheet dimensions of $20.3\times 25.4\;{\text{cm}}^{2}$. The film was handled according to the procedures described in the AAPM TG‐55 report.^(^ ^18^ ^)^

GAFCHROMIC EBT3 radiochromic dosimetry film is comprised of a single active layer, nominally 27 μm thick, containing the active component, marker dye, stabilizers, and other additives giving the film its low‐energy dependence. The yellow marker dye decreases UV/ light sensitivity and used in conjunction with an RGB film scanner, enables all the benefits of multichannel dosimetry. The active layer is between two, 120 μm transparent polyester substrates; this symmetric structure eliminates the need for keeping track of which side of the film is facing the light source of the scanner. The polyester substrate has a special surface treatment containing microscopic silica particles that maintain a gap between the film surface and the glass window in a flatbed scanner. Since the gap is nearly ten times the wavelength of visible light, formation of Newton's Rings interference patterns in images acquired using flatbed scanners is prevented.

### B. Radiochromic film calibration and irradiation procedures

Films were exposed in a phantom composed of $30\times 30\;{\text{cm}}^{2}$ sheets of solid water (PTW, Freiburg, Germany) with 10 cm of the buildup material above and below the film. The source‐to‐film distance was 100 cm. Film samples were cut ($10\times 12.5\;{\text{cm}}^{2}$) and irradiated perpendicularly to the 6 MV radiation beam from a dual‐energy Varian DHX‐S linac (Varian Medical Systems, Palo Alto, CA) equipped with a Millennium 120 leaf MLC. A $10\times 10\;{\text{cm}}^{2}$ field size at the isocenter was used.

A calibrated ion chamber PTW M30001 (PTW, Freiburg, Germany) was inserted in the phantom below the film plane to check the linac output during the irradiation process and to determine the dose delivered to the film by applying the IAEA‐TRS 398 protocol.^(^ ^19^ ^)^ To obtain a calibration curve, films were exposed at the dose levels of 0, 0.1, 0.3, 0.5, 0.7, 1, 1.5, 2, 2.5, 3, 3.5, 4, 5, 6, and 7 Gy.

### C. Scanning protocol and analysis

A flatbed scanner, Epson Expression 10000XL (Seiko Epson Corp., Nagano, Japan), and its associated software, EPSON SCAN v3.04, were used to read all the films.

To minimize the effect of the lateral dependence artifacts (the nonuniform response of the readout due to the light scattering of the scanner lamp caused by particles in the film active layer^(^ ^20^ ^)^, a $10\times 12.5\;{\text{cm}}^{2}$ cardboard template was fitted to the scanner to position films at a reproducible central location of the scan surface that can be considered uniform.^(^ ^16^ ^)^ To confirm this assumption, five OD measurements over the scanner central area were performed, resulting in a standard error less than 0.06%.

Images were acquired in transmission mode and landscape orientation, as recommended by the manufacturer, because the lateral response artifact on CCD scanners is smaller in this orientation compared to portrait orientation.^(^ ^20^ ^)^ RGB‐positive images were collected at a depth of 16 bits per color channel with a spatial resolution of 72 dpi corresponding to a pixel size of $0.35\times 0.35\;{\text{mm}}^{2}$, and saved in tiff format.

Image measurements and analysis were performed on a region of interest (ROI) of $1.5\times 1.5\;{\text{cm}}^{2}$. In addition, an opaque scan was obtained over the same ROI to account for dark current reading.

Raw images of irradiated films were imported from the scanning system into the RIT 113 v.5.2 analysis software (Radiological Imaging Technology, Inc., CO) for further image processing. To reduce inherent image noise, the software allows application of a 2D median filter of 5×5 pixels to the scanned film images.

The scanner response values were converted for every channel to net optical density (OD), and sensitometric curve was calculated using definitions given by Devic et al.^(^ ^21^ ^–^ ^22^ ^)^

The reproducibility of the flatbed scanner, obtained scanning repeatedly a film at different times, was below 0.2%. Film nonuniformity and film‐to‐film variations measured from three films from a single batch, following the method proposed by Saur et al.,^(^ ^3^ ^)^ were less than 1%.

The overall accuracy of EBT3 film measurements was derived using the method proposed by van Battum et al.^(^ ^23^ ^)^ that takes into account the most pronounced sources of uncertainties in dose determination (scanner, lateral correction, fit accuracy, intrabatch variation, background, intrinsic film inhomogeneity) and using error propagation analysis. An overall uncertainty of 1.7% was observed.

### D. Experiments

#### D.1 Response at high‐dose test

To investigate the film performance at high dose levels, 22 pieces of EBT3 film of $10\times 12.5\;{\text{cm}}^{2}$ were irradiated at different doses from 0 to 40 Gy. Net OD was obtained for every channel, and the sensitometric curves and their first derivatives were calculated.

#### D.2 Scanner orientation

The scan response of radiochromic films is sensitive to the orientation of the film on the scanner. This behavior results from the anisotropic scattering of the photons emitted by the scanner when passing through the polymer network, and the polarization of the transmit light by the needle‐like shape particles of film active component that are preferentially aligned parallel to the direction in which the film was coated that is parallel to the short edge of the film.

To estimate the effect of film orientation on scanner output for a given dose, eight of the calibration film pieces irradiated to the dose level of 0.3, 0.5, 1, 1.5, 2, 2.5, 3, and 4 Gy, were digitized on the scanning bed at 0° and 90°. Then the film pieces were flipped at the 180° orientation to examine any effects from film faceup versus facedown scan orientation. The net OD for each orientation was extracted from the $1.5\times 1.5\;{\text{cm}}^{2}$ ROI at the center of each image.

#### D.3 Postirradiation development with time

The EBT3 film postirradiation coloration, defined as the time in which variations produced in the color of the film are small enough to not lead to significant errors in its use for clinical applications, was evaluated scanning eight of the calibration film pieces irradiated to 0.3, 0.5, 1, 1.5, 2, 2.5, 3, and 4 Gy over a period of 72 h. For each film, the net OD was extracted from the $1.5\times 1.5\;{\text{cm}}^{2}$ ROI and used to construct a net OD growth curve as a function of time.

#### D.4 Energy dependence over IMRT range and dose rate response

To confirm the EBT3 film low‐energy dependence as specified by the manufacturer, eight film pieces were irradiated to the doses of 0.3, 0.5, 1, 1.5, 2, 2.5, 3, and 4 Gy with the photon energies generally used for IMRT treatments (6 MV and 15 MV X‐ray beams of the Varian linac).

The variation in film response due to different dose rate values (100, 300, and 600 MU/min) was studied for the same absorbed doses.

### E. Application to IMRT QA

To verify the possibility of scanning the EBT3 films from either side, an IMRT QA of a pelvis case was performed positioning the EBT3 film at 10 cm depth and SAD=100 cm inside a $30\times 30\times 20\;{\text{cm}}^{3}$ RW3 solid water phantom (PTW, Freiburg, Germany) in a plane perpendicular to the gantry rotation plane. Then the EBT3 film was scanned, varying the side facing the light source of the scanner but using the same calibration curve, and the relative dose maps were compared to each other and with the dose distribution calculated with the Oncentra MasterPlan version 4.1 TPS (Nucletron, Veenendaal, The Netherlands).

The irradiated films were scanned using the red color channel of the 48‐bit RGB mode (16 bits per color). The raw dose image was imported into the RIT 113 analysis software, calibrated and compared in absolute mode with the calculated dose map (1.5 mm resolution) using gamma analysis approach.^(^ ^24^ ^)^ A ROI encompassing the area within about 5 mm from the phantom edge was defined and the number of points satisfying the condition Γ<1 (Γ pass‐rate) was calculated, using different gamma evaluation criteria for dose difference (DD) and distance to agreement (DTA). The gamma calculation search radius was set to 1.0 cm. The DD criterion was calculated relative to the prescription dose. Points that lie outside the defined agreement tolerance can be easily distinguished on the compared dose map, and a histogram allows the user to know the percentage of points with a gamma value greater than 1.

To verify the response of the calibration procedure, a set of test intensity distributions for 6 MV highly modulated fields from the EBT3 film should be compared to EBT2 film directly. Γ index was calculated in absolute dose values, using 3%−3mm and 2%−1mm as gamma evaluation criteria.

Finally, to verify the feasibility of using the EBT3 GAFCHROMIC films in IMRT QA, the dose distribution of ten IMRT plans (five head and neck (H&N) and five pelvis) were measured with both EBT2 and EBT3, and the measured dose distributions were compared in absolute dose with those calculated, scaling the film dose to match the ionization chamber reading in the film plane and using 4%−3mm, 3%−3mm, and 2%−2mm as gamma evaluation criteria. Statistical significance of the differences between EBT3 and EBT2 response were determined using the Wilcoxon signed‐rank test for paired samples. Differences were considered significant for p<0.05.

## III. RESULTS & DISCUSSION

**Figure 1:**
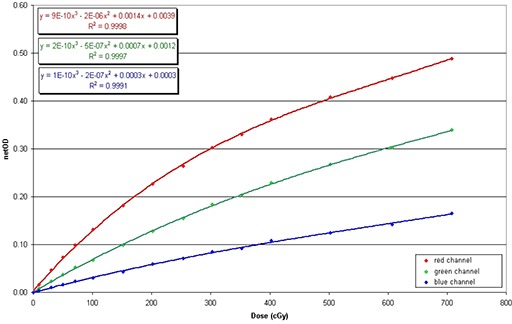
EBT3 film multichannel calibration curves up to 7 Gy.

The measured net OD corresponding to each channel determined from the pixel readings at different dose values employing the formulation described by Devic et al.^(^ ^21^ ^,^ ^22^ ^)^ are shown in Fig. 1. The sensitometric curves data were fitted with a third order polynomial. Fitting parameters and the agreement of the fit are also reported.

As illustrated, up to the net OD level of 7 Gy, the red channel showed a higher sensitivity than the green channel. Consequently, the red channel was used for the further image analysis.

### A. Response at high‐dose test

**Figure 2:**
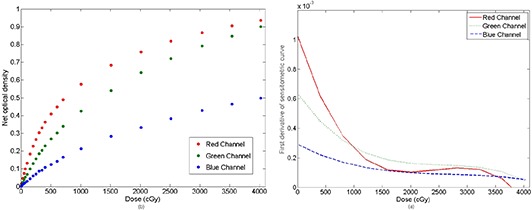
Net optical density vs. absorbed dose (a) and first derivative of sensitometric curves in the three channels of the film (b).

The dose response curves and their first derivatives for dose values up to 40 Gy are plotted in Fig. 2. The higher response (i.e., greater net optical density variation per unit dose) is reached in the curve whose derivative is higher. The difference in slope of the response curves has to be ascribed to the different ratio between the dose dependent and dose independent portion of the signal in each color channel.

The results confirm previous data published by Andres et al.^(^ ^7^ ^)^ As illustrated, the red channel has a greater response up to 10 Gy. The green channel exceeds the red one for doses above 10 Gy, indicating it could be preferable to use the green channel at higher doses. The blue channel had a lower response gradient at any dose because the signal has weak dose dependence while having strong dependence on the thickness of the active layer. This makes the blue channel less useful than the other channels for dose measurements.

It can be seen that first derivative of the sensitometric curves becomes negative from a dose level, different for each channel. The point at which the polynomial fit is no longer valid was calculated derived from the sensitometric function and looking for the absorbed dose value which makes null that derivative. This occurred at 38 Gy for the red channel and above 40 Gy for the green and the blue channels. Consequently, EBT3 can work up to nearly 40 Gy without a problem.

### B. Scanner orientation

**Figure 3:**
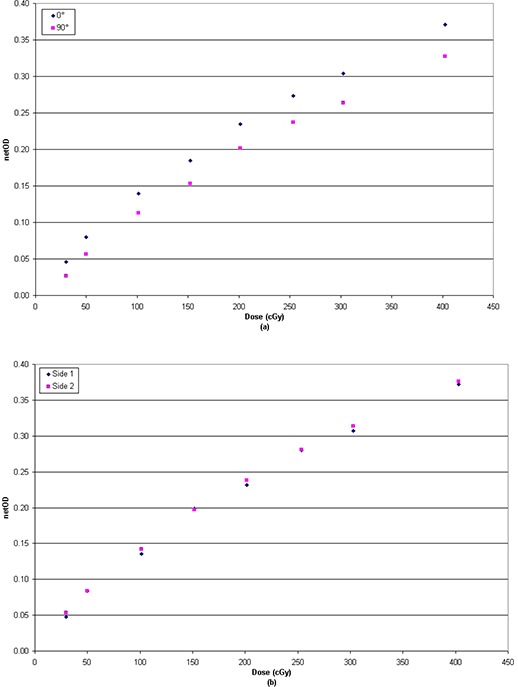
Sensitometric response (net optical density vs. dose) at different orientations of the film in the scanner.

Net OD obtained from every dose level varying the orientation of the film on the scanner is shown in Fig. 3. It can be seen that EBT3 film shows a difference up to 4.5% in net OD between portrait and landscape orientation (Fig. 3(a)).

Results showed lower dependence to those published for EBT2 by Andres et al.^(^ ^7^ ^)^ (∼7%−9%), although greater than that published by Desroches et al.^(^ ^6^ ^)^ (∼2%). Conversely, due to the symmetric structure of EBT3, differences from film faceup versus facedown scan orientation were negligible, with values less than 0.7% for doses up to 4 Gy (Fig. 3(b)). The side independent scanning can be considered the most important improvement of EBT3 film over EBT2, which showed a net OD difference between the two sides approximately equal to 2% which may significantly affect relative and absolute dose measurement.^(^ ^6^ ^)^

In practice, the EBT3 film can be scanned with either side facing the light source, but in the measurement and analysis of calibration and IMRT films, care must be taken not to mix films acquired in portrait orientation with those acquired in landscape.

### C. Postirradiation development with time

**Figure 4:**
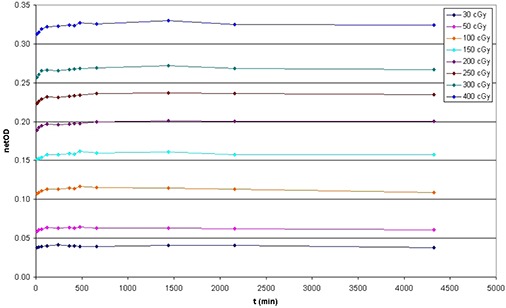
Change of the film coloration as a function of the time since irradiation for eight different dose levels.

**Figure 5:**
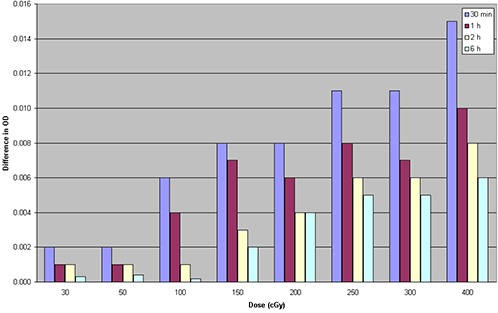
Variation of net optical density after 30 min, 1 h, 2 h, and 6 h compared to optical density 24 h after irradiation for eight different dose levels.

Net optical density vs. time after irradiation is illustrated in Fig. 4. In consideration of the importance to perform the IMRT QA analysis as soon as possible and the fact that between 24 h and 72 h after irradiation the variations in net OD were less than 0.005 for all doses under study, we compared the variation of net OD measured 30 min, 1 h, 2 h, and 6 h after irradiation to optical density 24 h after irradiation. The results are shown in Fig. 5.

As can be seen, EBT3 films show the same behavior as EBT2.^(^ ^7^ ^,^ ^13^ ^)^ In fact, between 1 h after irradiation and 24 h after irradiation, the variations in net OD were less than 0.010 for all doses under study, for variations between 2.1% and 4.3% in optical density. Between 2 and 24 h after exposure, changes in the net OD were less than 0.008, for variations in OD smaller than 2.5% at all doses in study.

It was observed that for doses less than 2 Gy, net OD seems to stabilize after nearly 30 min. Consequently, it could be possible to reduce the postirradiation stabilization period by reducing the irradiated dose.

In accordance with these results, the postexposure stabilization time of our measurement protocol for EBT3 film has been set in 2 h to guarantee the adequate stability to perform an analysis.

### D. Energy dependence over IMRT range and dose rate response

**Figure 6:**
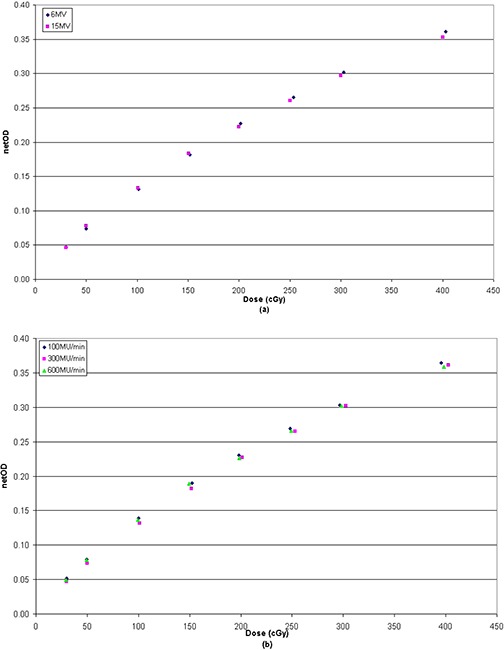
Energy (a) and dose rate (b) dependence of EBT3 film.

Net OD obtained varying the energy level and dose rate are shown in Fig. 6. Differences between films were negligible with values less than 1% for doses up to 4 Gy. The results show that EBT3 film response is nearly independent of radiation energy used in IMRT treatments, within the uncertainty of the measurement, and confirm previous data published for EBT2.^(^ ^10^ ^)^

### E. Application to IMRT QA

**Figure 7:**
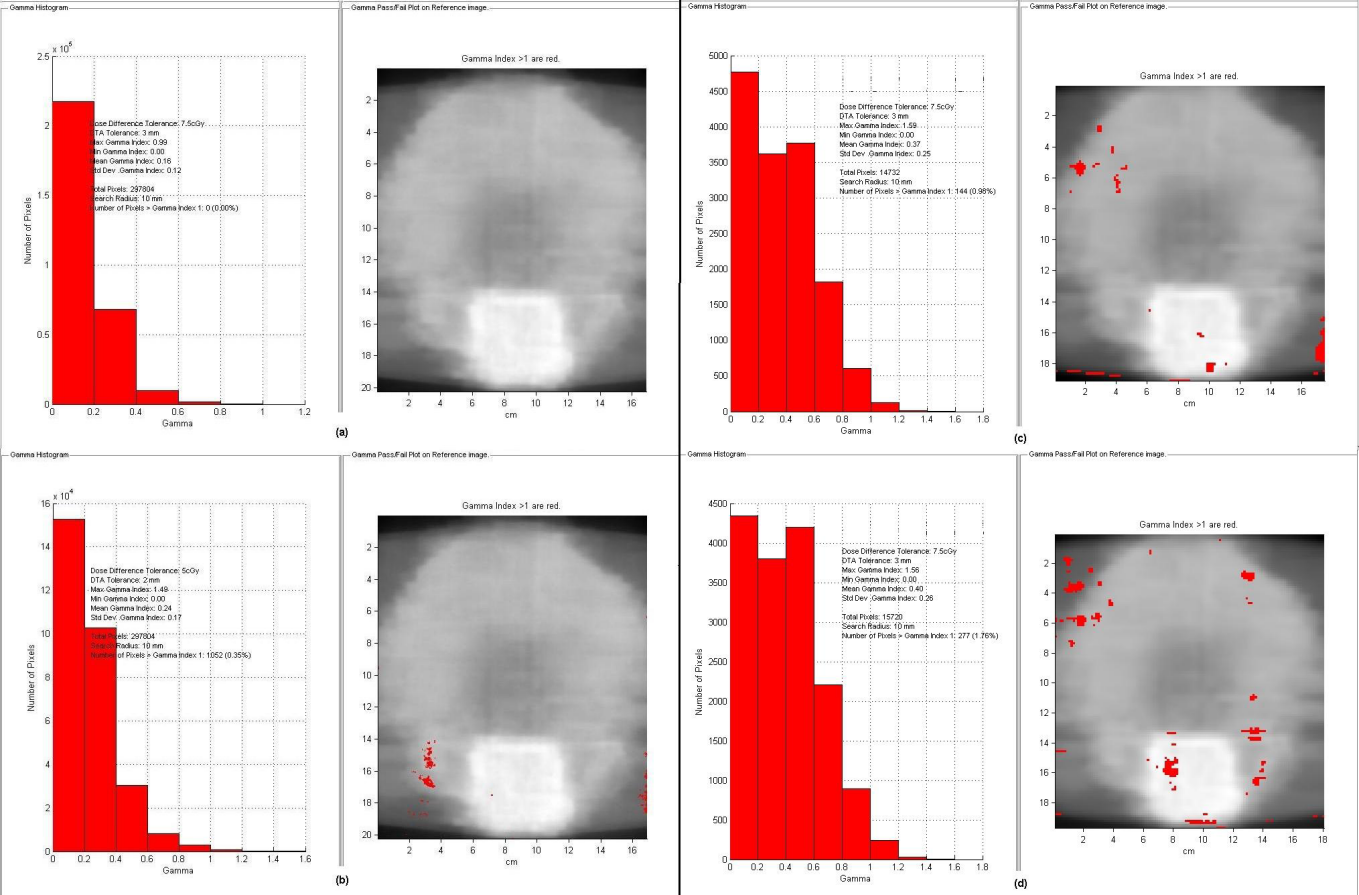
Γ analysis results of an example IMRT pelvis case obtained comparing the EBT3 dose maps scanned from both sides with the same calibration curve each other using 3%−3mm (a) and 2%−2mm (b) as DD and DTA criteria, and with the calculated dose maps (c) and (d) with 3%−3mm criteria.

Figure 7 shows the gamma index analysis for the IMRT pelvis case when the EBT3 film is scanned on sides 1 and 2 successively using the same calibration curve (i.e., that built from side 1 measurements) and the absolute dose maps are compared against each other. The two scanned dose maps were also compared with the calculated ones. As shown, also when using tighter gamma criteria (2%−2mm) almost all dose pixels passed the gamma test (99.7%).

The results confirm that to scan EBT3 film consistently on the same side is no longer as crucial as it was for the EBT2 films.^(^ ^6^ ^)^

**Figure 8:**
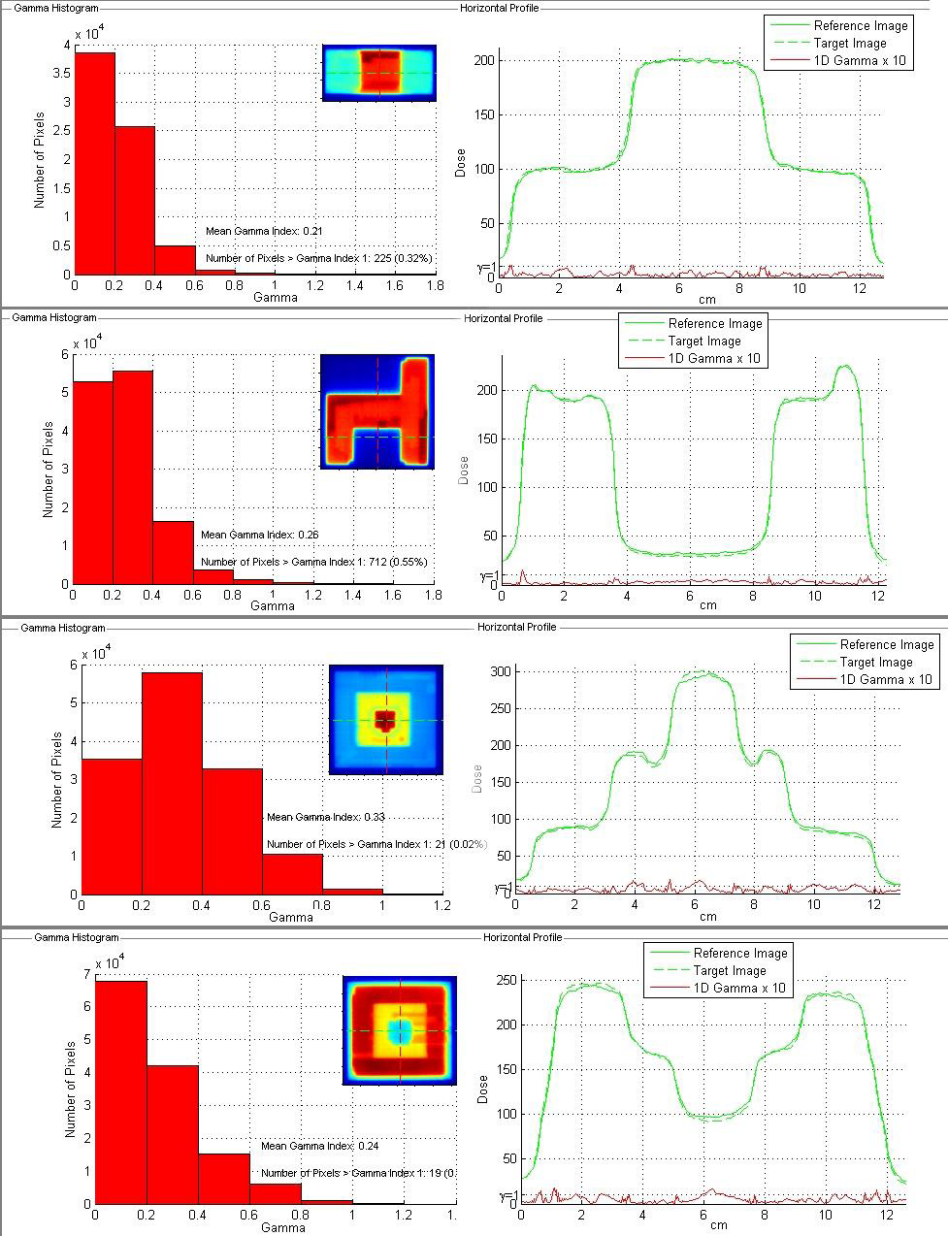
Statistical distribution of Γ index and EBT3‐to‐EBT2 dose profiles comparison for 6 MV highly modulated fields using 3%−3mm as DD and DTA criteria.

Figure 8 shows dose profiles and Γ histogram with 3%−3mm as DD and DTA constraints of the IMRT fields measured to evaluate the agreement between EBT3 and EBT2 film.

Statistical analysis shows that using a 3%−3mm criteria the percentage of points satisfying the Γ<1 condition is always more than 99%; when adopting a tolerance level of 2%−1mm, the Γ pass rate ranges from 92% to 96%, with the points not fulfilling the criterion Γ<1 usually located near the high‐dose gradient region.

**Figure 9:**
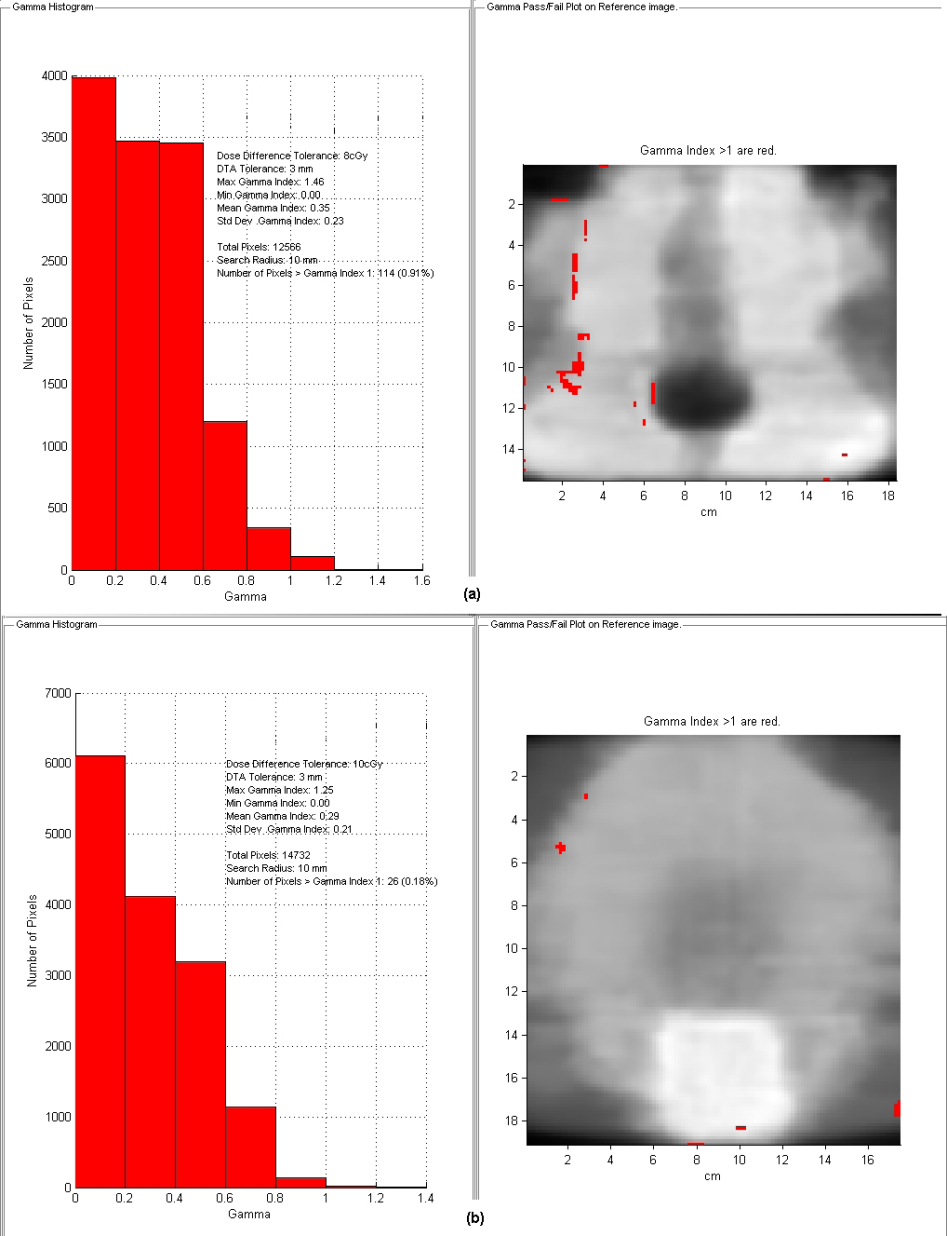
Γ value maps and Γ index statistics obtained with EBT3 film for a H&N (a) and a pelvis (b) plan using 4%−3mm as DD and DTA criteria.

In Table 1, the QA results of the ten IMRT cases are reported as mean value and standard deviation of the percentage of points satisfying the constraint Γ<1. Measurements of EBT3 films showed excellent agreement with the TPS calculations, with averaged fractions of passed Γ values greater than 99% and 97% using 4%−3mm and 3%−3mm gamma evaluation criteria, respectively, which are generally used in IMRT DQA applications. When the tightest criteria were used, an average Γ pass‐rate reduction of about 15% was observed. The percentage of points satisfying the constraint Γ<1 did not show significant differences between EBT2 and EBT3. In Fig. 9, gamma value maps and gamma index statistics are shown for a H&N and a pelvis plan. For these plans, the percentage of dose pixels passing the gamma test was 99.1% and 99.8%, respectively.

**Table 1:** Mean value and standard deviation of the percentage of points satisfying the constraint Γ<1 for each GAFCHROMIC film using different DD and DTA criteria. Statistically significant differences in the percentage of points satisfying the constraint Γ<1 between EBT3 and EBT2 were considered significant for p<0.05.

*GAFCHROMIC Film*	*Points with* Γ<1 *(%) [* 4%−3mm *]*	*p*	*Points with* Γ<1 *(%) [* 3%−3mm *]*	*p*	*Points with* Γ<1 *(%) [* 2%−2mm *]*	*p*
**EBT2**	98.9±0.6		97.2±1.1		84.3±3.6	
**EBT3**	99.3±0.3	NS	97.0±1.2	NS	82.4±3.9	NS

Notes: Two‐tailed *p* values from paired Wilcoxon test; data presented as mean ± standard deviation.

The similarity between EBT3 and EBT2 films based on the gamma analysis indicates that EBT3 film is a suitable alternative for routine IMRT QA to the well‐established EBT2 film.

## IV. CONCLUSIONS

The results of the analysis confirm the features introduced in the GAFCHROMIC EBT3 radiochromic film. Given that both films have the same composition of the sensitive layers, most of the characteristics of EBT3 film were found to be similar to the EBT2 film. The study of the colorization process shows a fast stabilization of the film within two hours. The color variation for unit dose has been investigated, finding that the red channel has a greater response up to 10 Gy, while the green channel is preferable at higher dose levels. The analysis varying the energy level and dose rate show no significant differences.

EBT3 film shows a different response between portrait and landscape orientation, but differences from film faceup versus facedown scan orientation are negligible.

The results of our investigation confirm that EBT3 film can be used for clinical practice in the same way as the previous EBT2 film. Moreover, the new enhancements make the EBT3 film more robust and easier to handle, making it applicable to replace the EBT2 film for IMRT dose verification.
